# Pathologic response of ductal carcinoma in situ to neoadjuvant systemic treatment in HER2-positive breast cancer

**DOI:** 10.1007/s10549-021-06235-2

**Published:** 2021-05-04

**Authors:** Emma J. Groen, Marieke E. M. van der Noordaa, Michael Schaapveld, Gabe S. Sonke, Ritse M. Mann, Mette S. van Ramshorst, Esther H. Lips, Marie-Jeanne T. F. D. Vrancken Peeters, Frederieke H. van Duijnhoven, Jelle Wesseling

**Affiliations:** 1 Department of Pathology, Netherlands Cancer Institute – Antoni van Leeuwenhoek, Plesmanlaan 121, 1066 CX Amsterdam, The Netherlands; 2 Division of Molecular Pathology, Netherlands Cancer Institute – Antoni van Leeuwenhoek, Amsterdam, The Netherlands; 3 Department of Surgical Oncology, Netherlands Cancer Institute – Antoni van Leeuwenhoek, Amsterdam, The Netherlands; 4 Division of Psychosocial Research and Epidemiology, Netherlands Cancer Institute – Antoni van Leeuwenhoek, Amsterdam, The Netherlands; 5 Department of Medical Oncology, Netherlands Cancer Institute – Antoni van Leeuwenhoek, Amsterdam, The Netherlands; 6 Department of Radiology, Netherlands Cancer Institute – Antoni van Leeuwenhoek, Amsterdam, The Netherlands; 7 Department of Radiology and Nuclear Medicine, Radboud University Medical Center, Nijmegen, The Netherlands; 8 Department of Internal Medicine, Onze Lieve Vrouwe Gasthuis, Amsterdam, The Netherlands; 9 Department of Pathology, Leiden University Medical Center, Leiden, The Netherlands

**Keywords:** HER2-positive breast cancer, Ductal carcinoma in situ, Response, Neoadjuvant systemic treatment

## Abstract

**Purpose:**

The presence of extensive ductal carcinoma in situ (DCIS) adjacent to HER2-positive invasive breast cancer (IBC) is often a contra-indication for breast-conserving surgery, even in case of excellent treatment response of the invasive component. Data on the response of DCIS to neoadjuvant systemic treatment (NST) are limited. Therefore, we estimated the response of adjacent DCIS to NST-containing HER2-blockade in HER2-positive breast cancer patients and assessed the association of clinicopathological and radiological factors with response.

**Methods:**

Pre-NST biopsies were examined to determine presence of DCIS in all women with HER2-positive IBC treated with trastuzumab-containing NST ± pertuzumab between 2004 and 2017 in a comprehensive cancer center. When present, multiple DCIS factors, including grade, calcifications, necrosis, hormone receptor, and Ki-67 expression, were scored. Associations of clinicopathological and radiological factors with complete response were assessed using logistic regression models.

**Results:**

Adjacent DCIS, observed in 138/316 patients with HER2-positive IBC, was eradicated after NST in 46% of patients. Absence of calcifications suspicious for malignancy on pre-NST mammography (odds ratio (OR) 3.75; 95% confidence interval (95% CI) 1.72–8.17), treatment with dual HER2-blockade (OR 2.36; 95% CI 1.17–4.75), a (near) complete response on MRI (OR 3.55; 95% CI 1.31–9.64), and absence of calcifications (OR 3.19; 95% CI 1.34–7.60) and Ki-67 > 20% in DCIS (OR 2.74; 95% CI 1.09–6.89) on pre-NST biopsy were significantly associated with DCIS response.

**Conclusions:**

As DCIS can respond to NST containing HER2-blockade, the presence of extensive DCIS in HER2-positive breast cancer before NST should not always indicate a mastectomy. The predictive factors we found could be helpful when considering breast-conserving surgery in these patients.

**Supplementary Information:**

The online version contains supplementary material available at 10.1007/s10549-021-06235-2.

## Background

Neoadjuvant systemic therapy (NST) that contains trastuzumab in addition to neoadjuvant chemotherapy leads to high pathologic complete response (pCR) rates in patients with human epidermal growth factor receptor 2 (HER2)-positive invasive breast cancer (IBC) [1–3]. Even higher pCR rates are seen when a trastuzumab-containing regimen is combined with the HER2-targeted antibody pertuzumab (i.e., dual HER2-blockade), with pCR rates of up to 80% reported in the HER2-positive/hormone receptor (HR)-negative subtype [4–9]. These excellent response rates allow for frequent conversion from mastectomy to breast-conserving surgery (BCS).

The presence of ductal carcinoma in situ (DCIS) adjacent to IBC, observed in 57–72% of HER2-positive breast cancer patients, may, however, impede this de-escalation of surgery, as DCIS is considered insensitive to systemic treatment [10–17]. A lower proliferative state, more intact physiological resistance mechanisms compared to IBC and a less receptive microenvironment to chemotherapeutic agents due to a protective basal membrane and less dense microvasculature have been put forward as potential causes for this therapy resistance [18–20]. Therefore the presence of a large area of calcifications on mammography or non-mass enhancement on MRI, both of which may be associated with DCIS, and/or extensive DCIS adjacent to IBC in pre-NST biopsies are often considered contra-indications for BCS, even in patients with radiological complete response of the tumor on breast MRI [21, 22].

However, data on the response of DCIS to NST are limited. A few studies have shown that DCIS may sometimes respond to NST [14, 23–25]. Two retrospective studies evaluating response of DCIS adjacent to HER2-positive breast cancer found that 36–51% of these DCIS lesions were eradicated after trastuzumab-containing NST combined with pertuzumab in a small subgroup [24, 25].

It is, however, not possible to predict which DCIS lesions adjacent to HER2-positive IBC will respond to NST. Imaging studies have difficulties to identify residual DCIS after NST, as the extent of calcifications on mammography after NST is very poorly associated with the pathologic response or residual size of invasive or in situ components [14, 16, 26, 27]. Therefore, performing BCS in patients with extensive DCIS is challenging, even when an excellent treatment response of their IBC has been achieved. To facilitate potential de-escalation of surgery in the future in this patient group, we aim to estimate the response of adjacent DCIS to NST containing HER2-blockade in a large series of HER2-positive breast cancer patients and to identify clinicopathological and radiological factors that predict response.

## Methods

### Patient and data collection

All women ≥ 18 years diagnosed with HER2-positive IBC who received NST containing HER2-blockade at the Netherlands Cancer Institute (NKI) between January 2004 and November 2017 were selected from the prospectively maintained NKI’s tumor registry.

Detailed patient, imaging, tumor and treatment characteristics were extracted from medical records. HER2 and HR status of IBC were assessed in all patients according to the Dutch guidelines. HR status was considered positive when ≥ 10% of luminal epithelial cells showed nuclear estrogen receptor (ER) expression, irrespective of progesterone receptor (PR) expression [22, 28]. Ki-67 in IBC was categorized into low (≤ 20% of expression) and high (> 20% expression) proliferation. Neoadjuvant chemotherapy regimens were categorized into taxane-based, anthracycline plus taxane-based or other. Type of HER2-blockade was registered (i.e., trastuzumab alone or dual HER2-blockade with trastuzumab and pertuzumab). Patients underwent both mammography and MRI pre-NST. All lesions were assessed by radiologists according to the BI-RADS lexicon [29]. For each tumor the size of the largest mass lesion, i.e., the index lesion, was reported as the largest diameter in the axial plane. In addition, the extent of the tumor was reported, being the size of the tumor area including surrounding satellites and non-mass enhancement. The presence and extent of calcifications suspicious for malignancy on pre-NST mammography was noted. A dedicated breast radiologist (RMM) reassessed mammographic images when relevant information regarding the presence or level of suspicion of calcifications (i.e., whether the calcifications were considered benign or suspicious for malignancy) was missing in the original report.

Tumor response was assessed on MRI after completion of NST, since MRI is superior to mammography in determining the presence and size of residual disease, and was categorized into (near) complete versus partial or no radiological response [30]. Radiological complete response was defined as no residual enhancement within the original tumor bed after NST. Near complete response was reported when only minimal residual enhancement (either some foci, or a diffuse glow) was visualized within the original tumor bed, without any components that were clearly identifiable as part of the original tumor. Post-NST mammography was not performed.

For women treated with breast-conserving surgery, the tumor was marked with a clip marker and localized with use of radio-guided occult lesion localization in the earlier years of our study cohort. In some patients, localization of the tumor was done with use of a wire. From 2007 the tumor was typically marked with an iodine seed prior to NST [31]. Breast-conserving surgery was planned using post-NST MRI findings. Specimen radiography was performed for all lumpectomies and for mastectomy specimens if a substantial pre-NST DCIS component was present to guide tissue sampling.

This study was approved by the institutional review board of the NKI.

### Pathology review

A dedicated breast pathologist (EJG) re-examined all pre-NST biopsies, blinded for response, to determine whether DCIS was present adjacent to IBC. These pre-NST biopsies mostly targeted the invasive component and were preferentially obtained under ultrasound guidance using a 14G core biopsy needle. In lesions that were ultrasound occult or presented as mammographic calcifications only, stereotactic biopsy was performed using a 9G vacuum needle. The number of available tissue cores was documented. If adjacent DCIS was present, the following histopathological DCIS features were scored: number of DCIS ducts, grade (1, 2 or 3) according to Holland criteria, dominant growth pattern (clinging, (micro-)papillary, cribriform, or solid), presence of calcifications, necrosis, periductal lymphocytic infiltrate, (type of) periductal fibrosis and mitotic activity (see scoring form in Supplementary methods) [32]. When slides originally stained with ER, PR, HER2 and Ki-67 contained DCIS, their expression was scored in the DCIS component (see details on antibodies in Supplementary methods). HER2 and HR status of DCIS were determined similarly as for IBC. As little is known about the distribution of Ki-67 in DCIS, Ki-67 in DCIS was categorized into two categories with the median used as cut-off value: low proliferation when ≤ 20% of cells showed expression and high proliferation when > 20% of cells showed expression.

Response of DCIS was defined as complete eradication of DCIS after NST. Data on the presence of residual DCIS in post-NST surgical specimens were retrieved from pathology reports. The number of slides that were originally examined was also noted. When no residual DCIS was described in the reports from women in whom adjacent DCIS was found in pre-NST biopsies, pathology slides were re-examined to affirm the eradication of DCIS.

### Statistical analysis

Descriptive statistics were used for patient, imaging, tumor and treatment characteristics. Included and excluded patients were compared, as were included patients with and without adjacent DCIS on pre-NST biopsy, using Pearson’s chi-squared test for categorical values and Wilcoxon rank-sum test or *t*-test for continuous variables. Adjacent DCIS was defined as any presence of DCIS on pre-NST biopsy.

Associations of clinicopathological and radiological factors with the response of DCIS to NST were assessed using logistic regression models. A stepwise regression was undertaken using forward selection. Variables were entered in multivariable models, based on a *P* value ≤ 0.05 in univariable analyses with elimination of variables at a threshold *P* value of > 0.05 in the multivariable analysis. Missing data on these eligible variables were imputed using chained equations (MICE) creating 50 datasets. Frequency of missingness was 1% for suspicious calcifications on mammography, 5% for tumor response on MRI, 5% for calcifications in DCIS in the biopsy, and 44% for Ki-67 expression in DCIS. Estimates from the imputed data sets were pooled using Rubin’s rule [33]. All tests were two-sided and *P* values ≤ 0.05 were considered statistically significant. All statistical analyses were performed using Stata/SE (version 13.1, Statacorp).

## Results

**Fig. 1:**
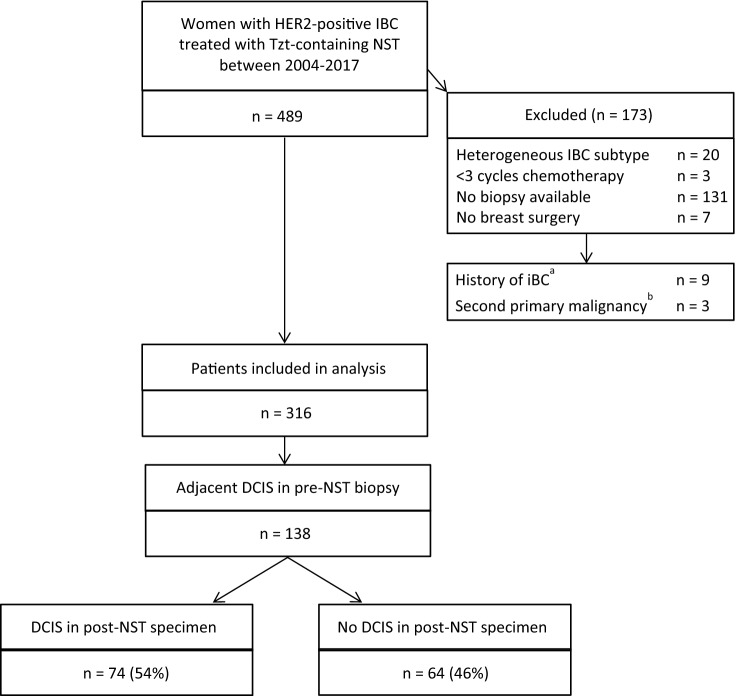
Flow diagram for patient selection and exclusions. *IBC* invasive breast cancer; *Tzt* trastuzumab; *NST* neoadjuvant systemic therapy; *n* number; *iBC* ipsilateral breast cancer. ^a^In situ and invasive breast cancer. ^b^Second primary malignancies, for which treatment may interfere with response evaluation of DCIS to NST

During the inclusion period, 489 patients with HER2-positive IBC received NST containing HER2-blockade at the Netherlands Cancer Institute. After exclusion of 173 patients, mainly because their pre-NST biopsies were not available for review (76%), 316 patients were available for further analyses (see flow diagram for patient selection and exclusions in Fig. 1). Included patients more often had lower-stage disease and were more frequently treated by a taxane-only regimen than excluded patients (see Supplementary Table 1, demonstrating clinicopathological characteristics of included and excluded patients).

Adjacent DCIS was observed in pre-NST biopsies from 138 out of 316 patients (44%). In 63 patients (20%) multiple biopsies were taken; in ten of these patients these biopsies targeted an area of calcifications or non-mass enhancement suspicious for an adjacent DCIS component. The remainder was targeted at the IBC only. Presence of adjacent DCIS increased with the number of examined tissue cores (*P* = 0.001), decreased with age (*P* = 0.047), was more frequent when suspicious calcifications were present on mammography (*P* = 0.005) and, in those with suspicious calcifications, increased when the extent of calcifications on the mammography was larger (*P* = 0.022; Table 1). Although patients with adjacent DCIS more often had a lower grade (grade 1 + 2 versus grade 3) of IBC, this association did not reach statistical significance (*P* = 0.054). At histopathological re-examination of pre-NST biopsies, DCIS was assigned grade 1 in 2% of patients, grade 2 in 45% and grade 3 in 53%. The HER2 status of DCIS could be assessed in 86/138 patients and was positive in 92%, equivocal in 7% (in these patients no SISH was available) and negative in 1% of patients. HR status of DCIS was positive in 63.5% and negative in 36.5% out of the 85 patients for whom HR stains were available. In 82% of these 85 patients, HR status of DCIS and IBC was concordant. In case of discordancy, a combination of HR-positive DCIS adjacent to HR-negative IBC was most frequently observed. In 9 out of 34 patients with HR-negative IBC the DCIS component was HR positive (26%), of these patients 67% showed a complete response (6/9 patients). Conversely, in the 6 (12%) out of 51 patients with HR-positive IBC with adjacent HR-negative DCIS, the response rate was 50%.

**Table 1:** Clinico-radiological and IBC factors in patients with and without adjacent DCIS

Factors	DCIS *n* (%)^a^ *n* = 138 (43.7)	No DCIS *n* (%) *n* = 178 (56.3)	*P*
Age at diagnosis, years, median (IQR)	45.9 (39.5–53.7)	48.6 (40.9–56.7)	0.047
Age at diagnosis			0.040
≤ 50 years	91 (65.9)	97 (54.5)	
> 50 years	47 (34.1)	81 (45.5)	
cT			0.54
T1	23 (16.7)	28 (15.8)	
T2	74 (53.6)	101 (57.1)	
T3	38 (27.5)	40 (22.6)	
T4	3 (2.2)	8 (4.5)	
cN			0.19
Node negative	50 (36.2)	52 (29.2)	
Node positive	88 (63.8)	126 (70.8)	
cM			0.19
M0	129 (93.5)	172 (96.6)	
M1	9 (6.5)	6 (3.4)	
Tumor size MRI before NST^b^			0.45
0–35 mm	73 (54.1)	88 (49.2)	
36–120 mm	62 (45.9)	89 (50.3)	
MRI size, mm, median (IQR)	34 (24–60)	36 (24–52)	0.66
Suspicious calcifications Mx			0.005
Absent	41 (29.9)	79 (45.4)	
Present	96 (70.1)	95 (54.6)	
Extent of suspicious calcifications^b^			0.031
5–55 mm	23 (41.8)	32 (62.8)	
56–140 mm	32 (58.2)	19 (37.3)	
Area suspicious calcifications, mm, median (IQR)	60 (35–88)	50 (20–70)	0.022
IBC subtype			0.003
No special type^c^	133 (96.4)	150 (84.8)	
Lobular	2 (1.5)	15 (8.5)	
Other	3 (2.2)	12 (6.8)	
Grade IBC^d^			0.054
Grade 1 + 2	71 (52.2)	70 (41.2)	
Grade 3	65 (47.8)	100 (58.8)	
HR status IBC			0.58
HR negative	60 (43.5)	83 (46.6)	
HR positive	78 (56.5)	95 (53.4)	
Ki-67 IBC, %			0.45
Low, ≤ 20	40 (39.2)	44 (34.4)	
High, > 20	62 (60.8)	84 (65.6)	
Chemotherapy			0.79
Taxanes	111 (80.4)	147 (82.6)	
Anthracyclines + taxanes	26 (18.8)	29 (16.3)	
Other	1 (0.7)	2 (1.1)	
HER2-blockade			0.37
Tzt	84 (60.9)	117 (65.7)	
Tzt + Ptz	54 (39.1)	61 (34.3)	
Type of surgery			0.11
Breast-conserving surgery	73 (52.9)	110 (61.8)	
Mastectomy	65 (47.1)	68 (38.2)	
Response on MRI			0.096
No/partial response	24 (18.3)	43 (26.5)	
(Near)complete response	107 (81.7)	119 (73.5)	

*IBC* invasive breast cancer, *n* number, *P* *P* value, *IQR* interquartile range, *NST* neoadjuvant systemic therapy, *Mx* mammography, *HR* hormone receptor, *Tzt* trastuzumab, *Ptz* pertuzumab

^a^One woman had bilateral breast cancer

^b^Tumor size on MRI before NST and extent of suspicious calcifications on mammography were categorized into two groups with the median in this group of 316 patients used as cut-off value

^c^Formerly known as invasive ductal carcinoma

^d^Grade IBC: only 1 patient had IBC grade 1 and did not have adjacent DCIS

Of the 138 patients with adjacent DCIS on pre-NST biopsy, 80% were treated with a taxane-based regime, 19% with an anthracycline plus taxane-based regime and in 1% with another regime. Sixty-one percent of patients received trastuzumab and 39% received dual HER2-blockade with trastuzumab and pertuzumab. A (near) complete radiological response on MRI was observed in 82% of patients. Seventy-seven patients were initially treated by lumpectomy and 61 by mastectomy. Resection margins were free in 87% of the women treated by breast-conserving surgery (67/77). Margins were involved in 10 patients due to irradically removed DCIS (*n* = 6), IBC (*n* = 1) or both (*n* = 3). Re-surgery was performed in 6 patients (re-lumpectomy in 2 and mastectomy in 4 patients) leading to a final free margin status. In the remaining 4 patients, who all showed only focally involved margins, no re-surgery was performed.

The median number of slides examined from post-NST surgical specimens for women with adjacent DCIS was 10 (interquartile range 8–14). After NST, DCIS was eradicated in 64 out of 138 patients (46%). The number of examined slides did not differ between patients with or without residual DCIS (*P* = 0.20). In 59% of patients who showed DCIS response, breast-conserving surgery was performed (without considering other pre-NST factors), while in the non-responder group this was 47% (*P* = 0.16). In women with residual DCIS after NST, DCIS was found without IBC in 39/74 women (53%; Table 2). In contrast, in women with residual IBC, IBC without DCIS was found only in 9 out of 44 patients (20%). Among the 178 patients in whom adjacent DCIS was not found on pre-NST biopsy, 61 patients (34%) had DCIS after NST based on pathology reports, which was associated with residual IBC in 38 patients (62%).

**Table 2:** Pathologic findings after NST in patients with and without DCIS in pre-NST biopsy

	DCIS in pre-NST biopsy *n* (%) *n* = 138 (43.7)		No DCIS in pre-NST biopsy *n* (%) *n* = 178 (56.3)	
	DCIS post-NST	No DCIS post-NST	DCIS post-NST	No DCIS post-NST
IBC post-NST	35 (47.3)	9 (14.1)	38 (62.3)	38 (32.5)
No IBC post-NST	39 (52.7)	55 (85.9)	23 (37.7)	79 (67.5)
Total *n*	74	64	61	117

*NST* neoadjuvant systemic therapy, *n* number, *IBC* invasive breast cancer

### Association between clinicopathological and radiological factors and response of DCIS to NST

The clinico-radiological factors, absence of suspicious calcifications on mammography (odds ratio (OR) 3.75; 95% confidence interval (95% CI) 1.72–8.17), treatment with dual HER2-blockade (OR 2.36; 95% CI 1.17–4.75) and a (near) complete response on MRI (OR 3.55; 95% CI 1.31–9.64) were associated with DCIS response in univariable analysis (Tables 3, 4), as were the histopathological factors absence of calcifications in DCIS on pre-NST biopsy (OR 3.19; 95% CI 1.34–7.60) and Ki-67 expression > 20% in DCIS (OR 2.74; 95% CI 1.09–6.89). Grade and HR status of IBC or DCIS was not associated with DCIS response. The number of patients with HER2-negative DCIS was too small to allow an informative analysis on the association of HER2 status in DCIS with treatment response.

**Table 3:** Associations of clinico-radiological and IBC factors with response^a^ of DCIS to NST in univariable analysis

Clinico-radiological factors	Total *n* (%)	Response *n* (%) *n* = 64 (46.4)	No response *n* (%) *n* = 74 (53.6)	OR^b^ (95% CI)^c^	*P*^d^
Age at diagnosis					
≤ 50 years	91 (65.9)	37 (57.8)	54 (73.0)	REF	
> 50 years	47 (34.1)	27 (42.2)	20 (27.0)	1.97 (0.97–4.02)	0.061
Chemotherapy					
Taxanes	111 (80.4)	50 (78.1)	61 (82.4)	REF	
Anthracyclines + taxanes	26 (18.8)	13 (20.3)	13 (17.6)	1.22 (0.52–2.87)	
Other	1 (0.7)	1 (1.6)		NA	0.65
HER2-blockade					
Tzt	84 (60.9)	32 (50.0)	52 (70.3)	REF	
Tzt + Ptz	54 (39.1)	32 (50.0)	22 (29.7)	2.36 (1.17–4.75)	0.015
Tumor size MRI before NST^e^					
7–34 mm	69 (50.0)	34 (53.1)	35 (47.3)	1.24 (0.63–2.44)	0.53
35–110 mm	66 (47.8)	29 (45.3)	37 (50.0)	REF	
Unknown	3 (2.2)	1 (1.6)	2 (2.7)		
Suspicious calcifications Mx					
Absent	41 (29.7)	28 (43.8)	13 (17.6)	3.75 (1.72–8.17)	
Present	96 (69.6)	35 (54.7)	61 (82.4)	REF	0.001
Unknown	1 (0.7)	1 (1.6)			
Extent of suspicious calcifications^e^					
13–60 mm	28 (29.2)	10 (28.6)	18 (29.5)	REF	
61–140 mm	27 (28.1)	11 (31.4)	16 (26.2)	1.24 (0.42–3.68)	0.70
Unknown	41 (42.7)	14 (40.0)	27 (44.3)		
Response on MRI					
No/partial response	24 (17.4)	6 (9.4)	18 (24.3)	REF	
(Near)complete response	107 (77.5)	58 (90.6)	49 (66.2)	3.55 (1.31–9.64)	0.008
Unknown	7 (5.1)		7 (9.5)		
IBC factors					
Grade					
Grade 1 + 2	71 (51.5)	37 (57.8)	34 (46.0)	1.63 (0.83–3.22)	
Grade 3	65 (47.1)	26 (40.6)	39 (52.7)	REF	0.16
Unknown	2 (1.5)	1 (1.6)	1 (1.4)		
HR status					
HR negative	60 (43.5)	32 (50.0)	28 (37.8)	1.64 (0.83–3.24)	0.15
HR positive	78 (56.5)	32 (50.0)	46 (62.2)	REF	
Ki-67, %					
Low, ≤ 20	40 (29.0)	18 (28.1)	22 (29.7)	REF	
High, > 20	62 (44.9)	30 (46.9)	32 (43.2)	1.15 (0.52–2.54)	0.74
Unknown	36 (26.1)	16 (25.0)	20 (27.0)		

*IBC* invasive breast cancer, *NST* neoadjuvant systemic therapy, *n* number, *OR* odds ratio, *CI*  confidence interval, *P* *P* value, *REF*  reference, *NA* not applicable, *Tzt* trastuzumab, *Ptz* pertuzumab, *Mx* mammography, *HR* hormone receptor

^a^Response is defined as complete eradication of DCIS after neoadjuvant systemic therapy

^b^Missings were not taken into account as a separate category

^c^Confidence interval is Wald-based

^d^*P* value is based on the LR-based test statistic

^e^Tumor size on MRI before NST and extent of suspicious calcifications on mammography were categorized into two groups with the median used as cut-off value

**Table 4:** Associations of DCIS factors with response^a^ of DCIS to NST in univariable analysis

DCIS factors	Total *n* (%) *n* = 138	Response *n* (%) *n* = 64 (46.4)	No response *n* (%) *n* = 74 (53.6)	OR^b^ (95% CI)^c^	*P*^d^
Grade^e^					
Grade 1 + 2	63 (45.7)	27 (42.2)	36 (48.7)	REF	
Grade 3	72 (52.2)	37 (57.8)	35 (47.3)	1.41 (0.71–2.78)	0.32
Unknown	3 (2.2)		3 (4.1)		
Growth pattern^f^					
(Non)solid	22 (15.9)	8 (12.5)	14 (18.9)	REF	
Solid	110 (79.7)	54 (84.4)	56 (75.7)	1.69 (0.66–4.34)	0.27
Unknown	6 (4.4)	2 (3.1)	4 (5.4)		
Calcifications					
Absent	99 (71.7)	55 (85.9)	44 (59.5)	3.19 (1.34–7.60)	0.006
Present	32 (23.2)	9 (14.1)	23 (31.1)	REF	
Unknown	7 (5.1)		7 (9.5)		
Necrosis					
Absent	69 (50.0)	39 (60.9)	30 (40.5)	1.98 (0.99–3.95)	0.053
Present	63 (45.7)	25 (39.1)	38 (51.4)	REF	
Unknown	6 (4.4)		6 (8.1)		
Mitoses					
Sparse	82 (59.4)	38 (59.4)	44 (59.5)	REF	
Many	48 (34.8)	23 (35.9)	25 (33.8)	1.07 (0.52–2.17)	0.86
Unknown	8 (5.8)	3 (4.7)	5 (6.8)		
Periductal fibrosis					
Absent + subtle	71 (51.5)	32 (50.0)	39 (52.7)	REF	
Prominent	53 (38.4)	27 (42.2)	26 (35.1)	1.27 (0.62–2.58)	0.52
Unknown	14 (10.1)	5 (7.8)	9 (12.2)		
Type fibrosis^g^					
Sclerotic	41 (46.1)	17 (42.5)	24 (49.0)	REF	
Myxoid	47 (52.8)	23 (57.5)	24 (49.0)	1.35 (0.58–3.15)	0.48
Unknown	1 (1.1)		1 (2.0)		
Lymphocytic infiltrate					
Absent + subtle	99 (71.7)	45 (70.3)	54 (73.0)	REF	
Prominent	27 (19.6)	14 (21.9)	13 (17.6)	1.29 (0.55–3.03)	0.56
Unknown	12 (8.7)	5 (7.8)	7 (9.5)		
HR status					
HR negative	31 (22.5)	15 (23.4)	16 (21.6)	1.17 (0.48–2.84)	0.73
HR positive	54 (39.1)	24 (37.5)	30 (40.5)	REF	
Unknown	53 (38.4)	25 (39.1)	28 (37.8)		
Ki-67, %					
Low, ≤ 20	39 (28.3)	14 (21.9)	25 (33.8)	REF	
High, > 20	38 (27.5)	23 (35.9)	15 (20.3)	2.74 (1.09–6.89)	0.030
Unknown	61 (44.2)	27 (42.2)	34 (46.0)		

*NST* neoadjuvant systemic therapy, *n* number, *OR* odds ratio, *CI* confidence interval, *P* *P* value, *REF* reference, *HR* hormone receptor

^a^Response is defined as complete eradication of DCIS after neoadjuvant systemic therapy

^b^Missings were not taken into account as a separate category

^c^Confidence interval is Wald-based

^d^*P* value is based on the LR-based test statistic

^e^Grade DCIS: only 2 patients had grade 1

^f^(Non)solid = clinging, (micro)papillary, cribriform

^g^Type of fibrosis was only scored when periductal fibrosis was present

All abovementioned, eligible factors except Ki-67 expression > 20% in DCIS, were also independently associated with DCIS response in multivariable analysis (see Supplementary Table 2). After multiple imputation, Ki-67 expression > 20% in DCIS no longer reached the significance level set for entry into multivariable analysis.

## Discussion

We have demonstrated that a part of the DCIS lesions adjacent to HER2-positive breast cancer can be eradicated after NST. To the best of our knowledge, this is the largest study that examined the response of DCIS, found adjacent to HER2-positive IBC, to NST containing HER2-blockade and the first study that assessed the association of clinicopathological and radiological factors with response. The response evaluation of adjacent DCIS is highly relevant, as NST containing HER2-blockade frequently results in pCR of HER2-positive IBC, but the presence of extensive, clinically detectable DCIS pre-NST often precludes performing BCS. Therefore, it would be most relevant to know in which patients adjacent DCIS will respond to NST to eventually increase the conversion rate of mastectomy to breast-conserving surgery. We have identified several factors associated with the response of DCIS to NST that can aid towards selection of a subgroup among HER2-positive breast cancer patients with extensive DCIS that could be treated by breast-conserving surgery.

In this study, we analyzed 316 women with HER2-positive IBC of whom 138 (44%) had adjacent DCIS in their pre-NST biopsies. Our incidence rate of DCIS was in the same range as reported by others who also evaluated the presence of adjacent DCIS in pre-NST biopsies, i.e., 37–46% in HER2-positive IBC [14, 24, 25]. However, a higher incidence rate of adjacent DCIS is seen in studies assessing its presence in surgical specimens of patients undergoing upfront surgery, i.e., 57–72% in HER2-positive IBC [13, 15, 17]. Our finding of residual DCIS after NST in 61 out of 178 patients (34%) without adjacent DCIS in their pre-NST biopsies underlines that identifying patients with adjacent DCIS in biopsies, targeting the invasive component, is less accurate.

Studies have suggested that IBC with adjacent DCIS is associated with less aggressive behavior compared to IBC without DCIS with significantly better overall survival (5-year overall survival, 89% versus 86%, *P* < 0.001) [13, 15]. Compared to IBC without DCIS, IBC with adjacent DCIS was associated with a lower Ki-67 expression and grade, ER/PR/HER2 positivity, lower tumor and nodal stage, and was more frequently found in pre-menopausal women [13, 15]. In our study, IBC with adjacent DCIS was associated with a younger age and the presence of suspicious calcifications on pre-NST mammography. In addition, DCIS was more often found adjacent to IBC grade 1 + 2, but this association did not reach statistical significance. Two other studies that evaluated the sensitivity of DCIS to NST did not find a correlation between the presence of adjacent DCIS and age, nodal status, IBC grade, HR status or Ki-67 [14, 24]. As these studies, like ours, were performed in women treated by NST partly focusing on HER2-positive IBC alone, and likely suboptimally identifying IBC with adjacent DCIS in pre-NST biopsies, associations may be different.

We found that DCIS was eradicated after NST in 64 out of 138 women with adjacent DCIS in their pre-NST biopsies (46%). Our results are in line with those of a smaller study by von Minckwitz et al., in which DCIS was eradicated in 30/59 patients (51%) with HER2-positive IBC who were treated with a neoadjuvant regimen including anthracyclines, taxanes and trastuzumab with or without capecitabine [24]. A slightly lower, but still comparable response rate of 36% was found in a study, which also focused on adjacent DCIS in HER2-positive IBC, in which patients were treated with taxane-based chemotherapy plus trastuzumab and also pertuzumab in a small subgroup [25]. Another study showed a pCR of DCIS, found adjacent to IBC of all subtypes, in 10 out of 30 patients (33%) treated with anthracycline–taxane-containing NST (plus trastuzumab when the HER2 receptor was overexpressed) [14].

Absence of suspicious calcifications on pre-NST mammography, dual HER2-blockade, a (near) complete response on MRI, the absence of calcifications in DCIS on pre-NST biopsy and a Ki-67 expression in DCIS of > 20% were associated with response of DCIS to NST in univariable analysis. The results for Ki-67 expression in DCIS should be interpreted with some caution due to the large proportion of missings. Reports on response of invasive HER2-positive breast cancer have identified similar factors, as complete response is more frequently observed in patients treated with dual HER2-blockade compared to trastuzumab alone, in patients with a (near) complete response on MRI or in IBC with a high Ki-67 expression [4–6, 8, 34, 35]. A recent review concerning HER2-positive IBC showed that three factors are associated with an increased pCR rate: (1) high HER2 combined with low estrogen receptor 1 gene expression levels, (2) a ‘HER2-enriched’ PAM50 intrinsic subtype, and (3) higher levels of tumor infiltrating lymphocytes [36]. Although we did not perform gene expression analysis, we evaluated HR status of IBC and DCIS, but did not find a higher response rate for HR-negative IBC or DCIS. It could be that response rates of HR-negative versus HR-positive DCIS does not parallel the situation for IBC in HER2-positive breast cancer patients. In our study cohort, women with HR-negative IBC did not differ from women with HR-positive IBC in terms of age, treatment, and grade or proliferation of IBC. There was a trend towards smaller tumor size in HR-positive IBC compared to HR-negative IBC based on T-stage and MRI size at baseline (*P* = 0.065 and *P* = 0.074, respectively), but this does not imply an association with a smaller size of the DCIS component per se. Perhaps a discordancy in HR status between DCIS and IBC may play a role here, but this seems unlikely when considering the small subset of such patients found in our cohort, of whom HR-negative IBC patients with adjacent HR-positive DCIS showed a higher response rate than HR-positive IBC patients with adjacent HR-negative DCIS (response rate 67% versus 50%). Lastly, HR status of DCIS was missing for 38% of all cases included that could mask an underlying difference in response rates between HR-positive versus HR-negative DCIS.

Our study has several limitations. One limitation is a lack of thorough radiological correlation with DCIS prior to NST, which would enable more accurate identification of patients with (extensive) DCIS, allowing for more accurate assessment of true response. A second limitation is intrinsic to the way in which IBC is diagnosed and classified prior to NST, i.e., by taking a biopsy targeted on the IBC and pathologic evaluation thereof. This implies that the aim of most biopsies is not to assess the presence of adjacent DCIS. This may compromise adequate evaluation of the response of DCIS to NST, as there is a risk of missing adjacent DCIS in pre-NST biopsies. Expanding our analysis by including patients who only showed (residual) DCIS after NST would enable rightful recognition of these ‘non-complete responders’. However, this would also lead to an underestimation of DCIS response because patients without DCIS in pre-NST biopsies who had a complete response would not be considered. In this context it is also important to note that in clinical practice DCIS can be occult on imaging, representing a subset of patients in whom adjacent DCIS was only identified after NST in our study. For these patients, prediction of DCIS response will not change surgical treatment decisions. A third potential minor limitation might be that the diagnostic biopsy procedure results in complete removal of a small component of adjacent DCIS, compromising response evaluation. Yet, as feasibility issues for breast-conserving surgery particularly arise in patients with extensive adjacent DCIS, it is unlikely that this will impact clinical practice.

In conclusion, we demonstrated in this exploratory study that complete response of DCIS to NST can be achieved in almost half of the patients with confirmed DCIS adjacent to HER2-positive IBC in pre-NST biopsies. Further research is needed to validate our findings within HER2-positive breast cancer patients with clinically detectable, extensive DCIS, while carefully correlating radiology and pathology of the DCIS component pre- and post-NST. Within such a context, the conversion rate of mastectomy to breast-conserving surgery, and recurrence and survival rates related to DCIS response could be evaluated. For now, our study indicates that the presence of extensive DCIS in HER2-positive breast cancer before NST should not always indicate a mastectomy, and the predictive factors we found could be helpful when considering BCS in these patients.

## Supplementary Information

Below is the link to the electronic supplementary material.

Supplementary file1 (DOCX 29 kb)

## Data Availability

The datasets generated during and/or analyzed during the current study are available from the corresponding author upon reasonable request. Requests should be made to Prof. Jelle Wesseling (j.wesseling@nki.nl).

## Abbreviations

NST: Neoadjuvant systemic therapy
pCR: Pathologic complete response
HER2: Human epidermal growth factor receptor 2
IBC: Invasive breast cancer
HR: Hormone receptor
BCS: Breast-conserving surgery
DCIS: Ductal carcinoma in situ
NKI: Netherlands Cancer Institute
ER: Estrogen receptor
PR: Progesterone receptor
OR: Odds ratio
95% CI: 95% Confidence interval
