# Supplementary material for: Pathologic response of ductal carcinoma in situ to neoadjuvant systemic treatment in HER2-positive breast cancer
Source: Breast Cancer Res Treat. 2021 May 4;189(1):213–24. doi: 10.1007/s10549-021-06235-2 (PMC8302531; doi:10.1007/s10549-021-06235-2)
Supplement: Supplementary file 1 — Supplementary file1 (DOCX 29 kb) [file 10549_2021_6235_MOESM1_ESM.docx]

**Supplementary methods**

**Scoring form for the evaluation of adjacent DCIS**

**DCIS:**

- absent
- present

**DCIS component:**

DCIS grade:

- 1: Well differentiated
- 2: Moderately differentiated
- 3: Poorly differentiated
- 4: not evaluable/interpretable

Dominant growth pattern DCIS:

- Clinging
- (Micro)papillary
- Cribriform
- Solid
- Not evaluable/interpretable

Calcification:

- Absent
- Present
- Not evaluable/interpretable

Necrosis:

- Absent
- Present
- Not evaluable/interpretable

Mitoses:

- Sparse
- Many
- Not evaluable/interpretable

Myoepithelial staining evaluable:

- Yes
- No

**Remarks**

**Number of biopsies available for revision:** ……………

**DCIS environment:**

Periductal fibrosis:

- Absent
- Prominent
- Subtle
- Not evaluable/interpretable

When fibrosis is present,

dominant type of fibrosis:

- Sclerotic
- Myxoid

Periductal lymphocytic infiltrate:

- Absent
- Prominent
- Subtle
- Not evaluable/interpretable

**Number of DCIS ducts:**

………..

**DCIS subtype:**

ER:

- Percentage: ___ %
- Not evaluable/done

PR:

- Percentage: ___ %
- Not evaluable/done

HER2 IHC:

- IHC score 0
- IHC score 1+
- IHC score 2+
- IHC score 3+
- Not evaluable/done

HER2 SISH:

- Not amplified
- Amplified
- Not evaluable/done

Ki-67:

- Percentage: ___ %
- Not evaluable/done

| **Primary antibody sources and dilutions** | | | |
| --- | --- | --- | --- |
| **Antigen** | **Clone** | **Dilution** | **Manufacturer** |
| ER | SP1 | Ready-to-use | Ventana Roche |
| PR | 1E2 | Ready-to-use | Ventana Roche |
| HER2 IHC | 4B5 | Ready-to-use | Ventana Roche |
| Ki67 | MIB1 | 1/100 | Agilent |
| HER2 SISH | INFORM Her2 DNA probe | Ready-to-use | Ventana Roche |
| ER = estrogen receptor; PR = progesterone receptor; HER2 = human epidermal growth factor receptor 2; IHC = immunohistochemistry; SISH = silver in situ hybridization | | | |

**Supplementary Table 1. Clinicopathological characteristics of included and excluded patients**

| **Clinicopathological characteristics** | **Included patients n(%) n=316 (64.6)** | **Excluded patients^a^ n(%)**  **n=173 (35.4)** | ***P*** |
| --- | --- | --- | --- |
| **Age at diagnosis, years, median (IQR)** | 47.5 (40.0-55.2) | 47.7 (39.4-53.8) | 0.66 |
| **Age at diagnosis** |  |  | 0.81 |
| ≤50 years | 188 (59.5) | 101 (58.4) |  |
| >50 years | 128 (40.5) | 72 (41.6) |  |
| **cT** |  |  | **0.010** |
| Tis |  | 3 (1.7) |  |
| T1 | 51 (16.2) | 24 (14.0) |  |
| T2 | 175 (55.6) | 86 (50.0) |  |
| T3 | 78 (24.8) | 43 (25.0) |  |
| T4 | 11 (3.5) | 16 (9.3) |  |
| **cN** |  |  | 0.069 |
| Node negative | 102 (32.3) | 42 (24.4) |  |
| Node positive | 214 (67.7) | 130 (75.6) |  |
| **cM** |  |  | 0.051 |
| M0 | 301 (95.3) | 157 (90.8) |  |
| M1 | 15 (4.8) | 16 (9.3) |  |
| **Histology IBC** |  |  | 0.35 |
| No special type^b^ | 283 (89.8) | 139 (93.9) |  |
| Lobular | 17 (5.4) | 5 (3.4) |  |
| Other | 15 (4.8) | 4 (2.7) |  |
| **Grade IBC** |  |  | 0.34 |
| Grade 1+2 | 141 (46.1) | 55 (51.4) |  |
| Grade 3 | 165 (53.9) | 52 (48.6) |  |
| **HR status IBC** |  |  | 0.53 |
| HR negative | 143 (45.3) | 74 (48.4) |  |
| HR positive | 173 (54.8) | 79 (51.6) |  |
| **Ki-67 IBC, %** |  |  | 0.30 |
| Low, ≤20 | 84 (36.5) | 23 (44.2) |  |
| High, >20 | 146 (63.5) | 29 (55.8) |  |
| **Chemotherapy** |  |  | **0.013** |
| Taxanes | 258 (81.7) | 118 (70.2) |  |
| Anthracyclines+Taxanes | 55 (17.4) | 46 (27.4) |  |
| Other | 3 (1.0) | 4 (2.4) |  |
| **HER2 blockade** |  |  | 0.36 |
| Trastuzumab | 201 (63.6) | 101 (59.4) |  |
| Trastuzumab+Pertuzumab | 115 (36.4) | 69 (40.6) |  |
| **Surgery** |  |  | 0.57 |
| Breast conserving surgery | 183 (57.9) | 90 (55.2) |  |
| Mastectomy | 133 (42.1) | 73 (44.8) |  |

n = number; ^a^ Patients excluded for reasons related to clinicopathological characteristics were not taken into account in the comparison between in- and excluded patients for these characteristics (f.e. patients excluded because they had invasive breast cancer with heterogeneity of HER2 and/or estrogen receptor were excluded in the comparison of histopathological characteristics of invasive breast cancer); *P* = P value; IQR = interquartile range; IBC = invasive breast cancer; ^b^ formerly known as invasive ductal carcinoma; HR = hormone receptor

**Supplementary Table 2. Associations of clinicopathological and radiological factors with DCIS response^a^ to NST in multivariable analysis**

| **Factors** | **Response n(%)**  **n=64 (46.4)** | **No response n(%)**  **n=74 (53.6)** | **OR (95%CI)^b^** | ***P*** | **OR (95%CI)^b^** | ***P*** |
| --- | --- | --- | --- | --- | --- | --- |
|  |  |  | ***univariable*** |  | ***multivariable*** |  |
| **HER2 blockade** |  |  |  |  |  |  |
| Tzt | 32 (50.0) | 52 (70.3) | **REF** |  | **REF** |  |
| Tzt + Ptz | 32 (50.0) | 22 (29.7) | 2.36 (1.17-4.75) | **0.016** | 3.29 (1.46-7.40) | **0.004** |
| **Suspicious calcifications**  **on Mx^c^** |  |  |  |  |  |  |
| Absent | 29 (44.8) | 13 (17.6) | 3.80 (1.75-8.29) |  | 3.51 (1.32-9.32) |  |
| Present | 35 (55.2) | 61 (82.4) | **REF** | **0.001** | **REF** | **0.012** |
| **Response on MRI^c^** |  |  |  |  |  |  |
| No/partial response | 6 (9.4) | 20 (27.3) | **REF** |  | **REF** |  |
| (Near)complete response | 58 (90.6) | 54 (72.7) | 3.63 (1.34-9.79) | **0.011** | 4.14 (1.36-12.59) | **0.012** |
| **Calcifications DCIS biopsy^c^** |  |  |  |  |  |  |
| Absent | 55 (85.9) | 50 (67.2) | 2.99 (1.26-7.09) | **0.013** | 4.17 (1.36-9.85) | **0.001** |
| Present | 9 (14.1) | 24 (32.8) | **REF** |  | **REF** |  |
| **Ki-67 DCIS biopsy^c^, %** |  |  |  |  |  |  |
| Low, ≤20 | 26 (40.4) | 43 (58.5) | **REF** |  |  |  |
| High, >20 | 38 (59.6) | 31 (41.5) | 2.09 (0.91-4.78) | 0.081 |  |  |

Variables were entered in multivariable models, based on a P value ≤0.05 in univariable analyses with elimination of variables at a threshold P value of >0.05 in the multivariable analysis.

^a^ response is defined as complete eradication of DCIS after neoadjuvant systemic therapy; NST = neoadjuvant systemic therapy; n = number; OR = Odds Ratio; CI = Confidence Interval; ^b^ ORs and 95% confidence intervals derived from pooled estimates from 50 imputed datasets; *P* = P value; REF = reference; Tzt = trastuzumab; Ptz = pertuzumab; Mx = mammography; ^c^ numbers based on average realizations in 50 imputed datasets
